# Maintaining the Phenotype Stability of Chondrocytes Derived from MSCs by C-Type Natriuretic Peptide

**DOI:** 10.3389/fphys.2017.00143

**Published:** 2017-03-08

**Authors:** Quan Shi, Zhiyong Qian, Donghua Liu, Jie Sun, Juan Xu, Ximin Guo

**Affiliations:** ^1^Department of Stomatology, Chinese People's Liberation Army General HospitalBeijing, China; ^2^Department of Advanced Interdisciplinary Studies, Institute of Basic Medical Sciences and Tissue Engineering Research Center, Academy of Military Medical SciencesBeijing, China; ^3^School of Biological Science and Medical Engineering, Beihang UniversityBeijing, China; ^4^Stomatology Center, General Hospital of Armed Police ForcesBeijing, China

**Keywords:** mesenchymal stem cells, chondrogenic differentiation, phenotype stability, C-type natriuretic peptide, hypothesis

## Abstract

Mesenchymal stem cells (MSCs) play a critical role in cartilage tissue engineering. However, MSCs-derived chondrocytes or cartilage tissues are not stable and easily lose the cellular and cartilage phenotype during long-term culture *in vitro* or implantation *in vivo*. As a result, chondrocytes phenotypic instability can contribute to accelerated ossification. Thus, it is a big challenge to maintain their correct phenotype for engineering hyaline cartilage. As one member of the natriuretic peptide family, C-type natriuretic peptide (CNP) is found to correlate with the development of the cartilage, affect the chondrocytes proliferation and differentiation. Besides, based on its biological effects on protection of extracellular matrix of cartilage and inhibition of mineralization, we hypothesize that CNP may contribute to the stability of chondrocyte phenotype of MSCs-derived chondrocytes.

## Introduction

Cartilage damage caused by the osteoarthritis (OA) can lead to chronic pain and disability. The global prevalence of knee OA was estimated to be 3.8% (2010), which brings a heavy burden to the society (Cross et al., 2014). Articular cartilage has a limited capacity for spontaneous repair after injury (Savkovic et al., 2014). Cartilage tissue engineering is considered more applicable for articular cartilage repair. Articular chondrocytes (ACs) and mesenchymal stem cells (MSCs; Hubka et al., 2014; Savkovic et al., 2014) are often employed as the seed cells in cartilage engineering. As a native and differentiated cell type, the application of ACs is limited because it is associated with dedifferentiation during expansion *in vitro* and limited donor tissue supply (Hong and Reddi, 2013; Hubka et al., 2014). MSCs, for example, bone marrow-derived MSCs, or adipose-derived MSCs, have the ability of differentiation into functional chondrocytes under appropriate culture conditions (Mazor et al., 2014). With many advantages, MSCs are widely used in the field of tissue engineering and being put prodigious faith by researchers.

However, accumulated evidences have indicated that the application of MSCs as seed cells for cartilage engineering is not yet as ideal as previously proposed. The chondrocytes and cartilage tissue obtained from MSCs are not stable and will easily lose their cellular and hyaline cartilage tissue phenotype during long time culture *in vitro* and transplantation *in vivo* (Tatebe et al., 2005; Pelttari et al., 2006; Farrell et al., 2014; Hubka et al., 2014). These phenotypic instability is characterized by an up-regulated expression of collagen type X (Col X), matrix metalloproteinase (MMP), and an increase in alkaline phosphatase (ALP) activity, which are all associated with osteogenic differentiation (Hubka et al., 2014). Switch from chondrogenic to osteogenic phenotype of the MSCs-derived cartilage results in loss of the physiological function of articular cartilage. Thus, how to maintain the stability of MSCs-derived chondrocytes phenotype is an important problem that needs to be solved.

Developmentally, transient cartilage is found in the cartilage anlage of endochondral bones (Delgado-Martos et al., 2013), such as the growth plate of long bones of the limbs. In this unique microenvironment, the mesenchymal stem cells undergo a series of special biological processes: condensation, overt differentiation of chondrocytes, proliferation, maturation, hypertrophy, and replacement of chondrocytes by osteoblasts. (Hall and Miyake, 2000; Tuan, 2006; Nguyen et al., 2016). Therefore, if out of the microenvironment of cartilage formation or the microenvironment was damaged, it is likely a natural propensity for the MSCs to differentiate into chondrocytes and then progress to hypertrophy, eventually ossified. In contrast, the chondrocytes in permanent hyaline cartilage on the articular joint surface would not develop to hypertrophy through lifespan. Thus, to make MSCs-derived cartilage as an available clinical therapy, measures must be taken to maintain the phenotype of MSCs-derived chondrocytes at the proliferating or prehypertrophic stage and prevent them from undergoing terminal differentiation to calcified tissue.

In order to solve this problem, some methods have been searched: co-culture of ACs and MSCs (Hubka et al., 2014), Hypoxia (Lee et al., 2013), supplement parathyroid hormone-related protein (PTHrP; Kim et al., 2008) or activation of TGFβ pathway (Craft et al., 2015), etc. However, ideal methods for maintaining the phenotype stability of MSCs-derived chondrocytes have not been established and proved clinically translatable, and each of the methods mentioned above has its disadvantages. For example, in order to co-culture of MSCs and ACs, it may require additional surgery and incision; Hypoxia is relatively difficult to implement; PTHrP and TGFβ pathway have extensive biological functions, which will affect other cells, tissues or organs. Besides, the main purpose of cartilage tissue engineering is to repair cartilage damage caused by OA, so it is important to find an effective solution to maintain the phenotype under the inflammatory microenvironment.

C-type natriuretic peptide (CNP) is one member of the natriuretic peptide family which consists of atrial natriuretic peptide (ANP), brain/B-type natriuretic peptide (BNP) and CNP (Olney, 2006). Different from the other natriuretic peptides, CNP mainly expresses in the growth plate of long bones limbs and plays a critical role in maintaining cartilage homeostasis through its effects on both chondrocyte proliferation and differentiation (Mericq et al., 2000; Prickett et al., 2005; Peake et al., 2014). Data from *in vivo* and *vitro* studies show that CNP and its receptor of natriuretic peptide receptor-B (NPR-B) can affect growth of cartilage, chondrogenic differentiation, and mineralization of the cartilage. Genetic mutations in CNP or NPR-B can lead to achondroplasia-like dwarfism in both mice and humans (Chusho et al., 2001; Nakao et al., 2015). CNP can stimulate chondrocytes proliferation and cartilage matrix production, down-regulates the expression of endochondral ossification markers (Waldman et al., 2008) and delay mineralization of tibia (Agoston et al., 2007). Even under the environment of inflammation, CNP can protect the cartilage matrix from degradation (Krejci et al., 2005). Therefore, CNP may play an important role in maintaining the stability of chondrocyte phenotype derived from MSCs.

## Hypothesis

For clinical application, a stable chondrogenic phenotype of MSCs must be achieved. Based on the previous reports, we hypothesize that CNP is potentially a candidate to maintain the stability of chondrogenic phenotype MSCs-derived chondrocytes.

## Evaluation of the hypothesis

### CNP promotes the cartilage development and chondrogenic differentiation

In mammals, the long bones of limbs are formed through endochondral ossification, which involves the conversion of an initial cartilage template into bone via proliferation, hypertrophy, cell death, and eventually ossified in the growth plate. The mRNA of CNP and NPR-B can be detected in the growth plate (Yamashita et al., 2000; Chusho et al., 2001) and the immunofluorescence also confined this (Olney, 2006). In animal models, mutation or knockout of CNP or NPR-B can lead to dwarfism (Komatsu et al., 2002; Tamura and Garbers, 2003); In contrast, ectopic CNP can rescue growth retardation in mouse model of achondroplasia (Yasoda et al., 2004).

In humans, genetic mutations of NPR-B can result in a disproportionate dwarfism, named acromesomelic dysplasia, Maroteaux type (AMDM) (Bartels et al., 2004). Conversely, skeletal overgrowth has been observed in patients with over-expression of CNP caused by a balanced translocation (Moncla et al., 2007). Moreover, the CNP analog has been used as a therapy for achondroplasia in some clinical trials (Legeai-Mallet, 2016).

*In vitro* studies have revealed that CNP can promote proliferation of primary chondrocytes (Waldman et al., 2008), as well as chondrocytes-derived from MSCs (Tezcan et al., 2010; Kocamaz et al., 2012). Woods et al. revealed that CNP could regulate cellular condensation of mouse embryonic limbs bud cells during micromass culture by increasing the expression of N-cadherin (Woods et al., 2007). Besides, CNP can stimulate cartilage extracellular matrix deposition. Supplement of 10 nM CNP increased accumulation of proteoglycans and collagen in the culture of chondrocytes (Waldman et al., 2008). By measuring the ^35^SO_4_, Mericq V et al. found that CNP increased the synthesis of glycosaminoglycan (GAG), one of the main cartilage matrix components (Mericq et al., 2000). Addition of CNP further increased the GAG synthesis in the human trabecular bone derived MSCs (Tezcan et al., 2010) and chicken bone marrow derived MSCs (Kocamaz et al., 2012).

What's more, in rat chondrosarcoma (RCS) chondrocyte model, CNP counteracts fibroblast growth factor 2 (FGF2) effects (which inhibit proliferation and trigger matrix degradation) by inhibiting the Erk pathway at the level of Raf-1. (Krejci et al., 2005; Pejchalova et al., 2007). Besides, CNP can activate protein kinase G (PKG) II and upregulate the synthesis of the chondrocyte extracellular matrix through as yet unknown mechanism (Pejchalova et al., 2007). Additionally, increased CNP expression can be stimulated by dexamethasone, which is routinely used for chondrogenic culture of MSCs (Agoston et al., 2006).

### CNP protects extracellular matrix of cartilage

CNP protects the RCS extracellular matrix from degradation by inhibiting the FGF2-mediated catabolic effects and partially antagonizes FGF2-induced expression, release and activation of MMP2, MMP3, MMP9, MMP10, and MMP13 (Krejci et al., 2005), which are all associated with the degradation of cartilage matrix. Manoj Ramachandran revealed that treatment with CNP can protect the cartilage matrix by reducing the release of nitric oxide (NO) and prostaglandin E2 (PGE_2_) and blocked catabolic effects induced by interleukin-1 β (IL-1β) in a dose-dependent manner (Ramachandran et al., 2011; Peake et al., 2013).

Even in the inflammatory environment, CNP overexpression in chondrocytes can turnover endochondral growth delay and inhibit cartilage damage in mouse model (Bukulmez et al., 2014). By culture of human chondrocytes in IL-1β conditioned medium *in vitro*, Peake et al. revealed that CNP can reduce inflammatory factor and hyaluronan production in human chondrocytes via members of the multidrug resistance protein (MRP) and diminish proinflammatory effects, suggesting that the CNP pathway is protective (Peake et al., 2015).

An appropriate microenvironment is important for the physiological activities of the cells. In the growth plate, chondrocytes undergo metabolic changes from proliferating to hypertrophic state and may also transdifferentiate to osteoblast-like cells (Yang et al., 2014). While in the proliferative zone, instead of becoming hypertrophy, or calcify. (Brighton, 1984) This zone composed large amount of extracellular matrix, such as proteoglycans. Besides, it was concluded that very little degradation of the extracellular matrix occurs in the proliferative zone. (Brighton, 1978, 1984) Considering that NPR-B mRNA is expressed primarily in these zones (Olney, 2006), CNP may play a critical role in maintaining the chondrocytes phenotype by balancing the rate of cartilage production of the extracellular matrix of the chondrocytes.

### CNP inhibits mineralization

As previously mentioned, Col X, ALP, MMP, and RANKL, etc., are the main markers associated with endochondral ossification. CNP can inhibit endochondral ossification by inhibition of the expression of these factors and maintain the chondrocyte phenotype. *In vitro*, CNP stimulation decreased the expression of Col X in chondrocytes (Waldman et al., 2008). Besides, in an organ culture model, CNP can delay tibia mineralization by down-regulating the Tnfsf11 gene that encodes RANKL (Agoston et al., 2007). RANKL is expressed in hypertrophic cartilage and it can stimulate the removal of hypertrophic cartilage by osteoclasts and facilitate vascular invasion and ossification (Xing et al., 2005). These remodeling events could be delayed after the expressing of RANKL was inhibited. Moreover, CNP may participate in the Indian hedgehog/Parathyroid hormone-related protein (Ihh/PTHrP) loop that inhibits prehypertrophic chondrocytes from entering the hypertrophic phase (Yamashita et al., 2000; Olney, 2006). The positive effects of CNP on maintaining the MSCs-Derived chondrocytes phenotype stability are summarized in Table 1.

**Table 1:** **Summary of the positive effects of CNP on maintaining the chondrocytes phenotype stability**.

**Objects**	**Treatment**	**Targeted gene/ protein/cytokine**	**Effects of CNP/NPR-B**
bovine ACs (Waldman et al., 2008)	Cells treated with CNP	proteoglycans and collagen ↑	• Promote proliferation • Promote ecm deposition
		type X collagen ↓	• Inhibit endochondral ossification
human ACs (Peake et al., 2015)	Cells treated with CNP	TNF-α, IL-1β, IL-8, L-10 and IFN-γ↓	• Protect cartilage matrix • Maintain cartilage homeostasis
human ACs (Ramachandran et al., 2011; Peake et al., 2013)	Cells treated with CNP	PKG II↑	• Promote matrix synthesis
		IL-1β↓	• Inhibit ECM degradation
RCS (Krejci et al., 2005)	Cells treated with CNP	MAPK/Erk pathway ↓	• Protect the ECM
			• Promote proliferation
		PKG II↑	• Promote ECM synthesis
mouse embryonic limb bud cells (Woods et al., 2007)	Cells treated with CNP	N-cadherin ↑	• Promote cell adhesion
		GAG and chondroitin sulfate↑	• Promote ECM synthesis
human trabecular bone MSCs (Tezcan et al., 2010)	Cells treated with CNP	GAG synthesis↑	• Promote chondrogenic differentiation
chicken bone marrow MSCs (Kocamaz et al., 2012)	Cells treated with CNP	GAG synthesis↑	• Promote chondrogenic differentiation
tibia organ culture (Agoston et al., 2007)	Organ treated with CNP	Tnfsf11 gene (encoding RANKL)↓	• Delay mineralization and cartilage remodeling
		gdf5 gene ↑	• Stimulate cell adhesion • Mediate the anabolic effects of CNP
		p38 ↑	• Promote proliferation and ECM synthesis in growth plate chondrocytes
tibia organ culture (Mericq et al., 2000)	Organ treated with CNP	^3^H-thymidine ↑	• Stimulate cell proliferation in the proliferative zone
		^35^SO4-GAG ↑	• Stimulate cartilage matrix production
mouse (Nakao et al., 2015)	CNP or GC-B knockout	SOX-9, type II collagen, Ihh↓	• Promote proliferation and differentiation
mouse (Bukulmez et al., 2014)	Arthritis model with CNP overexpressing and the chondrocytes	MAPK/Erk pathway ↓	• Prevent endochondral growth delay • protect against cartilage damage
		SOX-9 gene ↑	• Promote matrix synthesis • Maintain cartilage integrity
mouse (Chusho et al., 2001)	CNP knockout	cGMP↓	• Promote chondrocyte proliferation and differentiation
mouse (Yasoda et al., 2004)	CNP overexpression in achondroplasia mouse model	MAPK pathway ↓	• Rescues achondroplasia • promote ECM in the growth plate
human (Bartels et al., 2004)	Mutation of NPR-B	–	• Regulation of skeletal growth (NPR-B)

*↑, up-regulate, promote, activate or increase; ↓, down-regulate, inhibit, or decrease; RCS, rat chondrosarcoma; ACs, articular chondrocytes; ECM, extracellular matrix; GC-B, guanylyl cyclase-B; Ihh, Indian hedgehog homolog; GAG, glycosaminoglycan.X*.

## Conclusion

Phenotypic instability restricts the application of the MSCs as seed cells in cartilage engineering and an ideal solution has not been established. These previous studies indicate that CNP can promote the MSCs differentiate into chondrocytes at a higher efficiency compared to the traditional methods and it can increase the expression of related chondrogenic markers in a short term. However, none of these papers has long-term *in vitro* or *in vivo* studies to observe the effect of CNP on the stability of the MCS-derived chondrocytes/cartilage.

Based on available research results on CNP and MSCs studies and the developmental biology of cartilage and bone tissue, we propose that CNP may represent an important candidate as a regulator to maintain the chondrogenic phenotype of MSCs-derived chondrocytes as well as MSCs-based engineered cartilage both *in vitro* and *in vivo*. The potential mechanisms maybe: (1) CNP could down-regulate the expression of RANK and Col X to maintain the MSCs derived chondrocytes at the proliferating or prehypertrophic stage. (2) CNP could protect the cartilage matrix and inhibit calcification under the inflammation environment caused by the OA. Once verified, our hypothesis will likely direct the development of phenotypically more stable cartilage tissue for cartilage regeneration.

## Author contributions

Substantial contribution to the concept and design of this study: QS, JX, and XG; literature retrieval and information collection: QS, ZQ, DL, and JS; manuscript drafting: QS; manuscript revising: XG and JX. All authors read and approved the final manuscript.

## Funding

This study was supported in part by grants from the National Natural Scientific Foundation of China (NSFC No. 31170926 and No.81170523).

### Conflict of interest statement

The authors declare that the research was conducted in the absence of any commercial or financial relationships that could be construed as a potential conflict of interest.
